# Induction of Apoptosis in the Rat Bone Marrow Mesenchymal Stem Cells Following Sodium Arsenite Treatment with the Dose Lesser than that Used for Treatment of Malignant Patient

**Published:** 2012

**Authors:** Mohammad Husein Abnosi, Malek Solemani Mehranjani, Hamidreza Momeni, Elham Shojafar, Mozhgan Barati

**Affiliations:** 1 *Biology Department, Faculty of Sciences, Arak University, Arak 38156-8-8349, Markazi Province, Arak, Iran*

**Keywords:** Apoptosis, Mesenchymal stem cells, Rats, Sodium arsenite

## Abstract

**Objective(s):**Arsenic compounds are potent human carcinogen and produce a variety of stress responses in mammalian cells. Recently sodium arsenite has been recommended to be used as anti malignancy drug by American food and drug administration (FDA). In this study, we aimed to determine the apoptosis inducing effect of sodium arsenite on rat bone marrow mesenchymal stem cells exposed *in vitro*.

**Methodology:**Cell morphology was studied with the help of Hoechst and propidium iodide as well as with single cell gel electrophoresis(comet assay), TUNEL assay and caspase activity base on immunocytochemistry using commercial kit were considered to study the mechanism of cell death.

**Results:**Our result showed that the sodium arsenite with concentration of 0.1 µM in 36 hr induces caspase dependent apoptosis in rat bone marrow mesenchymal stem cells. This concentration is the lowest level of sodium arsenite to be reported with apoptosis induction ability in stem cells.

**Conclusion:**Since sodium arsenite is used in therapy, more research should be carried out on the effect of this chemical on stem cells, especially MSCs.

## Introduction

Arsenic is a naturally occurring element and a by-product of copper, lead and other metals smelters (1). It is a top environmentally hazardous substance (2) which was demonstrated to be a human carcinogen (3). Inorganic arsenic has been shown to freely cross the placenta and accumulate in the embryonic neuroepithelium (4). Arsenic exist in several oxidative states but its pentavalent (arsenate, As^+^^5^) and trivalent (arsenite, As^+3^) form are most prominent in the environment which have toxicological significance (2). The mechanism of arsenic trioxide (AS_2_H_3_) toxicity is not completely understood but disruption of ion homeostasis with subsequent hemolytic action of sodium arsenite cytotoxic effect due to production of reactive oxygen species have been reported (5). In addition arsenite, having a high affinity for thiol groups in proteins, can form complexes with thiol group of cysteine and inhibit more than 200 enzymes (6). 

Based on the FDA recommendation arsenic trioxide (arsenite) has been used for the treatment of relapsed or refractory of acute promyelocytic leukemia in 2000 (7, 8). In addition there are reports indicate that the concentration of sodium arsenite less than 5 µM which is appear to be presented in the serum of chemotherapy patients, slow down the cell cycle progression through each cell cycle phase in U937 cell without decreasing cell viability (9, 10). Yadev and *et al* in their report showed that only high concentration (>5 µM) but not low concentration (<5 µM) of sodium arsenite has effect on viability, DNA synthesis, morphology, cell cycle and apoptosis of human mesenchymal stem cells (11). Whereas our previous study showed that the different dose of sodium arsenite (0.1, 0.5, 2.5, 12.5 and 20 µM) in 36 hr caused significant reduction of rat bone marrow mesenchymal stem cells (rMSCs) viability in culture media (12). Mesenchymal stem cells posses two fundamental characteristics: the ability of extensive replication and the capacity of multilineage differentiation among bone, cartilage and adipose cell lineages (13, 14). Presence of sodium arsenite in the serum would bring its toxicity to the bone marrow of the patients, and the mesenchymal stem cells as a good source to replenish the other cells (adipocytes, osteoblasts and chondrocytes) would be in great danger. Thus in this study we aimed to pinpoint the mechanism of cell death in rMSCs treated with 0.1 µM sodium arsenite for 36 hr, as at this concentration a highly significant (*P*< 0.001) reduction in the viability of the rMSCs was reported (12). 

## Materials and Methods

***Cell*** ***culture***

Six to eight week male Wistar rats of the albino strain were killed using diethyl ether and under sterile condition their femora and tibias were removed surgically, then using flashed out technique the bone marrow content were extracted in 3 ml of Dulbecco modified Eagle medium (DMEM) supplemented with 15% FBS and penicillin/streptomycin. The bone marrow content was centrifuged at 2500 rpm for 5 min at room temperature and pellet of the cells were homogenized with 1ml fresh culture media and transferred in a culture flask. After 24 hr, unattached cells were washed off the flask with PBS+ (containing Mg^++^ and Ca^++^) and adherent fibroblast-like cells were allowed to grow for 10-14 days, with every three days of culture media replacement. Cells were passaged at 90% confluence by trypsinization (Trypsin/EDTA solution; sigma) and reseeded at a density of 10^6^ cells in T_75 _plastic flask up to 3rd passage (15).

***Exposure to sodium arsenite***

Sodium arsenite were purchased from Merck Company (Germany). After 24 hr of plating the rMSCs into the plastic flask (to conform the cell attachment), the cells were exposed to 0.1 µM of sodium arsenite for 36 hr. 

***Morphology***

The attached rMSCs in a 24-well plate were treated with 0.1 µM of sodium arsenite in culture media for 36 hr. Chromatin staining was performed with Hoechst (H) to detect nuclear morphology in addition to that, the diameter of the nuclei of the control and treated cells were measured in µm with the help of Motic Image Software (micro optical group company version 1.2). Propidium iodide (PI) was used to counter stain the cells along with Hoechest and also to differentiate live cells from dead ones, in addition to sutdy the morphology of the cytoplasm, acridine orange (AO) was used. The cells were observed using fluorescence microscopy (Olympus, IX70).

***Single cell gel electrophoresis***

DNA breakage was observed using single-cell gel electrophoresis (comet assay) as described by Lynn *et al* (16) with some modification. Briefly, rMSCs after sodium arsenite treatment were harvested and embedded in 1٪ low melt agarose (Fermentas company) gel at a density of 1×10^6^ cells/ ml, and spread on a microscopic slide previously coated with normal melting point agarose. The slides were immersed in ice-cold lysis buffer (10 mM Tris-HCl, 2.5M NaCl, 100 mM Na_2_EDTA, 1٪ sodium N-lauryl sarcosinate, pH 10) for 1 hr at 4 ºC. Cellular DNA was denatured in electrophoresis buffer (300 mM NaOH AND 1 mM Na_2_EDTA) for 20 min at room temperature then electrophoresis was performed for 20 min at constant voltage (25 V). All the procedure was carried out under indirect light, and then the slides were washed in distilled water and renatured in 0.4 M Tris-HCl (pH 7.5). The slides were stained with ethidium bromide (2 µg/ml) and examined under the fluorescence microscope (Olympus, BX51).

***TUNEL assay***

Treated rMSCs in 12 well-plates were fixed using 4٪ paraformaldehyde in PBS for 1 hr at room temperature. Then nuclear DNA fragmentation was detected by terminal deoxynucleotidyl transferase mediated dUTP nick end labeling (TUNEL) method using the *in situ* cell death detection kit ( Roche, Germany, Catalog #: 11684817910) according to company instruction and visualized under the light microscope.

***Activated caspase-3 examination***

rMSCs were seeded at a density of 1×10^5^ cells/ml in a 12 well-plate and incubated in the presence or absence of 0.1 µM sodium arsenite for 36 hr. Then activated caspase-3 enzyme was examined using a fluorogenic substrate assay kit (Chemicon, Germany, Catalog #: APT403) according to company instruction and visualized under the fluorescence microscope (Olympus, IX70). To visualize the activated caspase-3 under the light microscope, the cells were then stained and counterstained according to the manufacturer’s protocol with DAB and hematoxylin respectively. Then the cells were observed under a light microscope equipped with a digital camera with 20X magnification.

***Statistical analysis***

Statistical evaluation of the data was performed using t-test. Results were shown as mean±S.D and *P*< 0.05 was accepted as the minimum level of significance.

## Results

***Morphological study***

**Figure 1:**
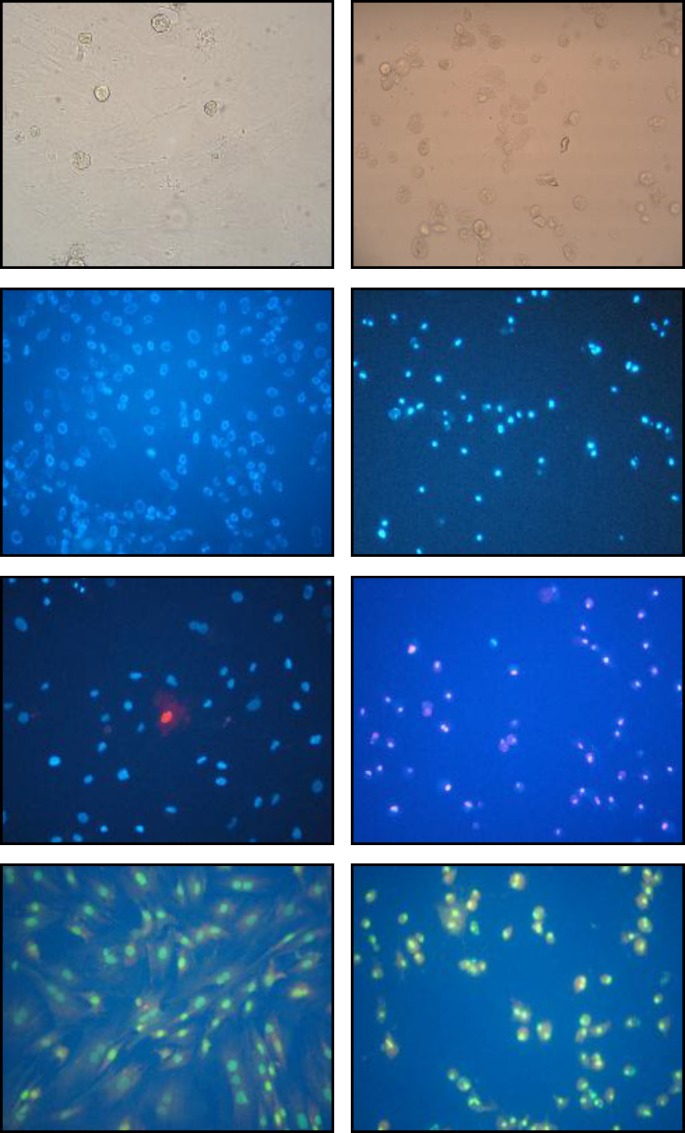
Morphology of the cells A) control cells without staining, monolayer of cells were seen B) sodium arsenite treated cells without staining, cells with round cytoplasm, losing their attachment to the bottom of the flask C) control cells stained with Hoechst, nuclei of the untreated cells appear large and no nuclear breaking was observed D) sodium arsenite treated cells stained with Hoechst, nuclei appear small as compared with control one and also nuclear breakage was observed E) co-staining of control cells with Hoechst and propidium iodide, only few cells are stained in red F) sodium arsenite treated cells stained with Hoechst and propidium iodide, most of the nuclei appear red in color which indicate the cell membrane damage G) control cells stained with acridine orange, typical morphology of the mesenchymal cell cytoplasm was visualized H) sodium arsenite treated cell stained with acridin orange, cell cytoplasm shrinkage and roundness was observed.

Using Hoechst fluorescent dye we showed that 0.1 µM of sodium arsenite after 36 hr caused chromatin condensation and nuclei shrinkage (Figure 1) as well as significant reduction (*P*< 0.001) in nuclei diameter (Nuclei diameter of 6.41± 0.90 and 3.48±0.90 µm for control and treated cells respectivly). In addition with the help of PI as counter stain, elevated number of cell death were observed. Morphology of treated rMSCs cytoplasm using light and florescent microscope (with the help of acridine orange as florescent dye) showed cytoplasm shrinkage, roundness of the cells and detachment of the cells from the bottom of the culture flask (Figure 1). 

***Single cell gel electrophoresis***

**Figure 2:**
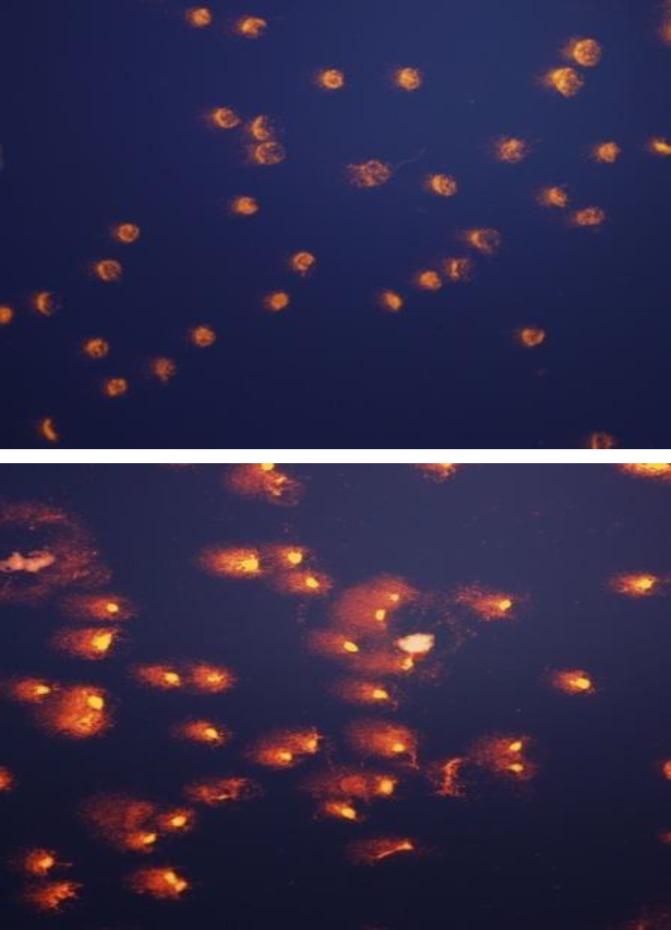
Comet assay A) control cells, no breakage in the DNA of the cell were observed B) sodium arsenite treated cells, due to DNA breakage a comet like tail has appeared behind the remaining part of the nuclei.

A very sensitive method called "single-cell alkaline gel electrophoresis" or "comet assay" in a quantitative level was adopted to investigate the state of the DNA. This method showed that the treatment of rMSCs with 0.1 µM sodium arsenite for 36 hr caused the DNA of the cell to break in large pieces in which a tail is formed behind the cells to indicate the comet formation (Figure 2). 

***TUNEL assay***

**Figure 3:**
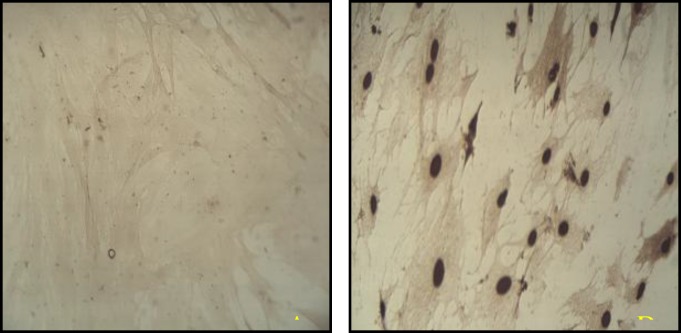
TUNEL assay A) control cells, no brown color nuclei is observed B) sodium arsenite treated cells, most of the cell nuclei stained brown which indicated the chromatin breakage in the cell nuclei.

To ascertain apoptotic changes in the rMSCs, TUNEL staining was performed and TUNEL-positive rMSCs after treatment with 0.1 µM of sodium arsenite for 36 hr were observed (Figure 3).

***Immunocytochemistry of activated caspase***

**Figure 4:**
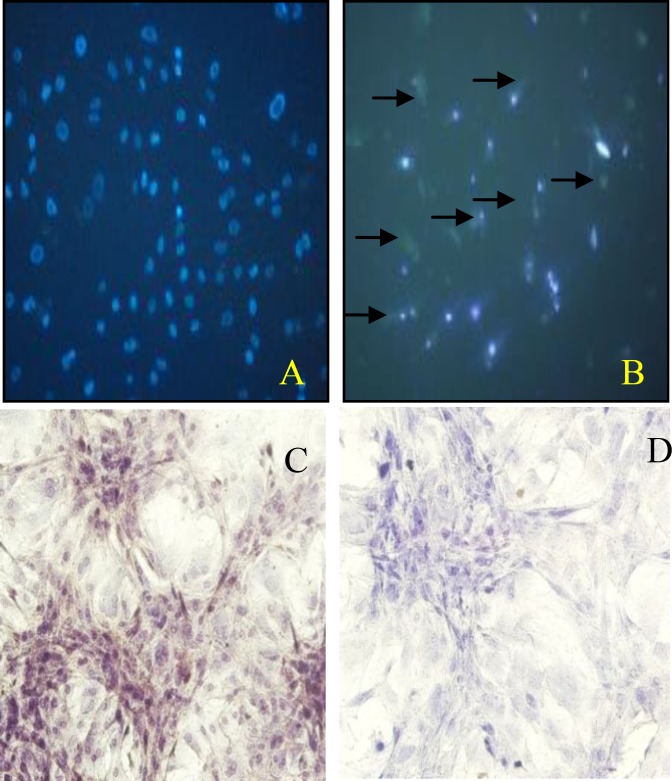
Activated caspase-3 assay A) control cells, no sign of green florescent in cytoplasm was detected B) sodium arsenite treated cells; most of the cell cytoplasm showed green florescent (arrows) C) control cells co-stained with DAB and hematoxylin, no brown color in the cytoplasm D) sodium arsenite treated cells co-stained with DAB and hematoxylin; most of the cell cytoplasm were showing brown in color to indicate the presence of the activated caspase- 3.

Immunocytochemistry staining showed that the rMSCs treated with 0.1 µM of sodium arsenite for 36 hr were having activated caspase- 3 in their cytoplasm (Figure 4).

## Discussion

Our previous study showed that the treatment of the rMSCs with 0.1 µM of sodium arsenite for 36 hr reduce viability of these cells significantly (12). As it is bvious in the Figure 1-F, the reduction in the viability of treated cells was also confirmed by staining them with combination of Hoechst and propidium iodide, where the numbers of red nuclei appear to be more than control one. Propidium iodide is highly hydrophobic in nature and cannot pass through the intact cell and nuclei membrane of the cells, but if there is any alteration in the integrity of the cell membrane, this fluorescent dye would pass through the cell membrane and bound to the chromatin of the cell which stains them in red (17). There are many reports that indicate the sodium arsenite reduction of the cell viability *in vitro* ( 18-20 ), also recently it was reported that the sodium arsenite with concentration more than 5 µM reduce the viability of human bone marrow mesenchymal stem cells (hMSCs) (11). Whereas in our report the 0.1 µM could reduce the viability of rat bone marrow mesenchymal stem cells significantly, the differences in the results might be due to the origin of the cells (18). The next challenge to be answered was the type and the mechanisms of cell death in rMSCs due to sodium arsenite toxicity. Thus the morphological study of the nuclei with the help of Hoechst and acridine orange fluorescent dye showed the condensation of chromatin, significant reduction of nuclear diameter and shrinkage of the cytoplasm which are the typical indication of apoptosis (4). Cytoplasmic shrinkage may be caused by cytoskeleton changes due to the effect of sodium arsenite on polymerization of tubulin monomers (21). Also nuclear breakage (21) and condensation (22) can be formed due to the caspase activity in treated cells with sodium arsenite. Thus in the next step, TUNEL assay showed that the DNA breakage has happened in the treated cells, moreover, sensitive method of comet assay confirmed that the broken large pieces of DNA have form a tail behind the cell under the electrical field. Previous studies showed that 5 µM arsenite significantly induces chromatin breaking in human fibroblasts (23) and hMSCs (11) which confirm our study on rMSCs. As necrosis also causes positive TUNEL and DNA breakage (24) thus the immunoreactivity of activated caspase-3 was investigated. Caspase-3 is activated in the apoptotic cell both by extrinsic and intrinsic pathways (25), thus activation of caspase-3 in the cells treated with 0.1 µM of sodium arsenite showed the happening of caspase dependent programmed cell death. Therefore the mechanism of cell death by low concentration of sodium arsenite is caspase dependent apoptosis as it was reported by Yadav *et al* for higher concentration of this chemical (11).

## Conclusions

These studies contributed to our understanding of how environmental chemicals (even at low concentration) induce apoptosis. Sodium arsenite which recently was recommended as a potent drug for treatment of malignancy, inappropriately activate apoptotic pathways leading to reduction of viability of rMSCs. Thus using chemical agents as medicine might have drastic effect on the other stem cells and itself is the causative reason of other health related problems. Therefore base on the result of this study, more research should be carried out on the effect of sodium arsenite on stem cells, especially on MSCs with respect to low dose and long term exposure as well as origin of these cells. 
